# Anxiety Suppressed Prefrontal Cortex Brain Activity: Insights From a Large Sample of Functional Near‐Infrared Spectroscopy (fNIRS) Data

**DOI:** 10.1155/da/9910013

**Published:** 2026-03-03

**Authors:** Honglin Ren, Yajie Wang, Youcai Yang, Qiang Xiao, Yan Zhang, Fuxing Wang, Hui Shi, Marc N. Potenza, Delong Zhou

**Affiliations:** ^1^ School of Psychology, Central China Normal University, Wuhan, China, ccnu.edu.cn; ^2^ Department of General Education, Wuhan Vocational College of Software and Engineering, Wuhan, China, whvcse.com; ^3^ Department of Psychology and Behavioral Sciences, Zhejiang University, Hangzhou, China, zju.edu.cn; ^4^ Key Laboratory of Adolescent Health Assessment and Exercise Intervention of Ministry of Education, East China Normal University, Shanghai, China, ecnu.edu.cn; ^5^ College of Physical Education and Health, East China Normal University, Shanghai, China, ecnu.edu.cn; ^6^ Department of Neurology, Hospital of Huazhong University of Science and Technology, Wuhan, China, hust.edu.cn; ^7^ School of Education, Center for Teaching Creative and Critical Thinking, Huazhong University of Science and Technology, Wuhan, China, hust.edu.cn; ^8^ The Department of Cardio-Psychiatry Liaison Consultation, Beijing Chao-Yang Hospital, Capital Medical University, Beijing, China, ccmu.edu.cn; ^9^ School of Medicine, Yale University, New Haven, USA, yale.edu; ^10^ Business School, Durham University, Durham, UK, dur.ac.uk

**Keywords:** anxiety severity, EAMT, fNIRS, Oxy-Hb, prefrontal cortex, right frontopolar cortex

## Abstract

**Background:**

Anxiety is one of the most common mental disorders and is linked to alterations in prefrontal cortex (PFC) function. Research on the underlying neuroscience has significant theoretical and practical implications. This study used a more naturalistic task and a larger sample to clarify how anxiety relates to brain activity.

**Materials and Methods:**

We recruited 841 participants and grouped them by Hospital Anxiety and Depression Scale (HADS) anxiety scores into no anxiety (NA), suspected anxiety (SA), and confirmed anxiety (CA). During an emotional autobiographical memory task (EAMT), a 53‐channel functional near‐infrared spectroscopy (fNIRS) system measured oxyhemoglobin (Oxy‐Hb).

**Results:**

Group differences were most pronounced in Channels 30 and 33 within the right frontopolar cortex (rFPC): NA and SA showed higher Oxy‐Hb than CA, whereas NA and SA did not differ (*p* < 0.001 for NA/SA > CA). Emotional valence showed no main or interaction effects. Oxy‐Hb in rFPC correlated negatively with anxiety severity (Ch30: *r* = −0.15, *p* < 0.001; Ch33: *r* = −0.16, *p* < 0.001), with similar patterns across additional channels.

**Conclusions:**

rFPC hypoactivation differentiates clinically significant anxiety from lower‐symptom groups and scales with symptom severity during EAMT. Findings support fNIRS as a practical physiological index for characterizing anxiety‐related prefrontal dysfunction in large samples. Future work should incorporate short‐separation channels and broader diagnostic measures.

## 1. Introduction

Anxiety is a feeling that occur when the source of harm is uncertain or is distal in space or time [1], and anxiety disorder is one of the most common mental challenges throughout life, causing considerable emotional and physical distress, as well as imposing substantial social and economic burdens [2–4]. Moreover, anxiety disorder patients typically exhibit attention, interpretation, and (sometimes) memory biases toward threat, and examining memory biases in patients with anxiety disorders is crucial, as intrusive memories are a common symptom in various anxiety disorders [5].

Autobiographical memory (AM) encompasses specific episodic memories of past events and more general self‐related information. Over‐general AM (OGM) is a notable aspect of AM, characterized by difficulty in recalling specific memories (lasting <24 h) and instead recalling general memories [6, 7]. To study OGM, the AM test (AMT), originally developed by Williams and Broadbent [8], has been employed as a standard measure for memory specificity. AMT, widely employed in affective science, is an emotion induction technique that utilizes written emotional memories to evoke emotions [9, 10]. Most neuroimaging studies employ symptom‐provocation paradigms contrasting negative versus neutral/positive stimuli [11, 12]. During the AMT, participants are presented with cue words (e.g., “feast” and “sad”) and asked to recall a specific memory related to each cue, and AMT biases play a role in the development and maintenance of anxiety [13]. Therefore, using the AMT task to induce brain activation is a feasible approach to exploring the brain activity patterns of individuals with different levels of anxiety.

Brain imaging studies are crucial for advancing our understanding of the neural circuits involved in anxiety disorders [12, 14]. Functional near‐infrared spectroscopy (fNIRS) is a newly developed optical neuroimaging technology characterized by noninvasiveness, affordability, portability, and ease of operation. It utilizes light sources with spectral windows between 650 and 1000 nm to penetrate organic tissue spectroscopy, primarily measuring hemodynamic changes, specifically changes in oxyhemoglobin (Oxy‐Hb) and deoxyhemoglobin (Deoxy‐Hb) concentrations near the brain’s surface [15, 16]. fNIRS has been proposed as a potential diagnostic method for detecting a variety of mental disorders, including anxiety [17]. Compared to other neuroimaging techniques, fNIRS monitors brain hemodynamic changes in a natural state and is less sensitive to motion artifacts. Therefore, this technology eliminates the discomfort of immobilizing participants in a confined space within an MRI/PET scanner, making it particularly suitable for individuals who may feel anxious or restless in their surrounding environment [15, 18].

Previous studies have shown that rises in Oxy‐Hb and reductions in Deoxy‐Hb detected using fNIRS are markers of cortical activation [19, 20]. Prefrontal cortex (PFC) is a crucial area in anxiety studies [14, 21]. Multiple neuroimaging studies on anxiety have been conducted; however, these results are inconsistent [12]. One possible reason for this inconsistency is sampling bias due to small sample sizes. Therefore, this study aims to conduct a large‐sample investigation to explore the brain activity patterns of individuals with varying levels of anxiety. In summary, this study aimed to explore the differences in brain hemodynamic activation among anxiety subgroups with varying levels of symptom severity, as well as the correlation between symptom severity and specific brain hemodynamic concentration. The study utilized the Hospital Anxiety and Depression Scale (HADS) as a screening measure for anxiety, combined with fNIRS technology, and involved a large sample of over 800 individuals. We hypothesized that the brains of individuals in the anxiety group would be less easily activated, and that higher anxiety scores would be associated with lower concentrations of Oxy‐Hb.

## 2. Materials and Methods

### 2.1. Participants

A total of 841 university students were recruited from the Psychiatry Department of Huazhong University of Science and Technology Hospital in Wuhan, China, between September 2019 and October 2023. The overall sample consisted of 464 males and 377 females (mean age ± SD of 21.48 ± 3.26; range = 17–35 years), 413 participants (49.1%) were undergraduates and 428 (50.9%) were graduate students. Participants were stratified into three anxiety‐based subgroups: 321 with no anxiety (NA), 307 with suspected anxiety (SA), and 213 with confirmed anxiety (CA), detailed demographic characteristics are presented in Table 1.

**Table 1:** Demographic and clinical data across three groups (M ± SD).

Variables	NA (*n* = 321)	SA (*n* = 307)	CA (*n* = 213)	*F* /*χ^2^*	*p*
Age	21.30 ± 3.38	21.36 ± 3.10	21.94 ± 3.25	2.87	0.057
Gender	—	—	—	7.70	0.021
Male	191 (59.63%)	172 (56.03%)	101 (47.42%)	—	—
Female	130 (40.37%)	135 (43.97%)	112 (52.58%)	—	—
Education level	—	—	—	3.95	0.139
Undergraduate	133 (41.61%)	106 (34.53%)	74(34.74%)	—	—
Graduate	188 (58.49%)	201 (65.47%)	139 (65.26%)	—	—
Anxiety score	5.73 ± 1.18	8.70 ± 0.71	14.43 ± 2.34	2327.10	<0.001
Depression score	6.60 ± 2.00	8.05 ± 1.99	9.77 ± 2.58	138.86	<0.001

All participants were right‐handed, native Chinese speakers with no history of psychotropic medication use and were independently screened by three senior psychiatrists. Exclusion criteria included: (1) left‐handedness; (2) current or past psychiatric or neurological disorders; (3) substance use or addictive disorders; (4) major medical illnesses; and (5) noncompliance with cognitive task requirements. Ethical approval was obtained from the Ethics Committee of Huazhong University of Science and Technology (Approval Number: 20190912), and all participants provided written informed consent before the study.

### 2.2. Measures

Prior to their fNIRS monitoring, all subjects underwent assessment using the HADS. The HADS is a 14‐item self‐report measure consisting of seven items each for depression and anxiety subscale, developed by Zigmond and Snaith [22]. Each item is rated on a 4‐point scale from 0–3, with 3 indicating a higher frequency of symptoms. The cumulative score for anxiety subscale varies between 0 and 21. Based on the HADS‐anxiety scores, participants were divided into NA group (range: 0–7), SA group (range: 8–10), and CA group (range: 11–21).

### 2.3. Emotional Autobiographical Memory Task (EAMT)

Numerous scholars have expanded the traditional AMT paradigm beyond words, and confirmed the usefulness of images as AM cues [23]. To effectively evoke the emotions of the participants, we substituted emotional pictures for words in this study, using the emotional autobiographical memory task (EAMT). The EAMT has been used successfully in both clinical and subclinical anxiety samples to induce reliable cortical responses without undue stress or attrition [24, 25].

The six cue pictures (positive, neutral, and negative) used in the EAMT were selected from the Chinese Affective Picture System (CAPS), which has been standardized in university samples and demonstrates high reliability even among individuals with elevated anxiety [26].

**Figure 1:**
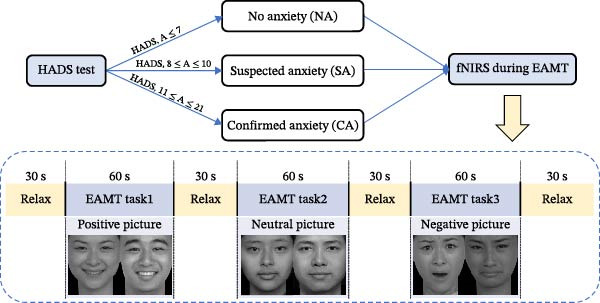
Experimental procedure.

During the EAMT, participants viewed the emotional pictures presented through PowerPoint in a randomized order. Voice instructions preceded each picture presentation, and participants were instructed to recall specific events related to each picture as quickly and detailedly as possible. The task consisted of three stages: a 30‐s pre‐task rest, a 180‐s EAMT task with three recall periods (positive, negative, and neutral cues), and a 30‐s post‐task rest (Figure 1). Participants were not allowed to communicate during the task.

### 2.4. fNIRS Measurement

**Figure 2:**
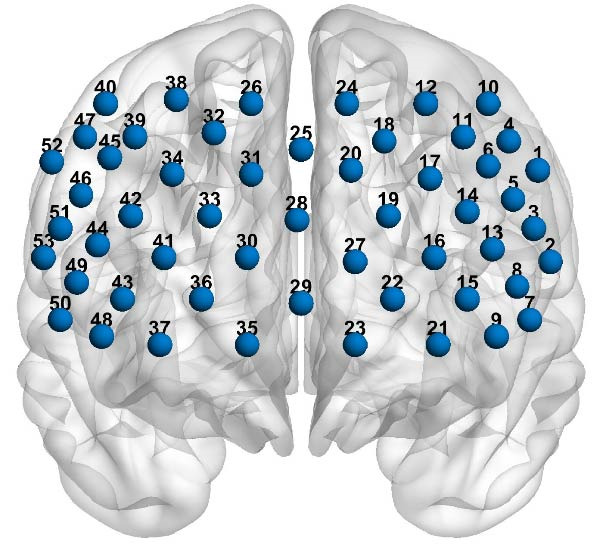
Locations of the 53 channels for the functional near‐infrared spectroscopy (fNIRS).

A 53‐channel fNIRS device (BS‐7000, Wuhan Znion Technology Co., Ltd., China) was used to monitor oxyhemoglobin (Oxy‐Hb) concentration changes. The device was equipped with 16 pairs of emission and detector probes that emitted light at wavelengths of 760 and 850 nm, operating at a frequency of 15.625 Hz. The distance between each emitter and detector falls within the range of 2.9–3.1 cm. Each probe was positioned on the forehead scalp, following the optode arrangement based on the 10/20 System of Electrode Placement commonly used in EEGs [27]. The arrangement of 53 channels is depicted in Figure 2. To normalize the fNIRS channel, we applied a 3D digitizer (NirMap, Wuhan Znion Technology Co., Ltd, Wuhan, China) to record the exact spatial coordinates of four reference points (Nz, Cz, AL, and RL) and 33 probes. Subsequently, the 53 channels were converted to an estimated Montreal Neurological Institute (MNI) space [28] using the probabilistic registration method by NIRS‐SPM [29, 30]. Detailed mappings are provided in Supporting Information: Appendix S1.

### 2.5. Statistical Analysis

Prior to acquisition, only participants who passed device calibration (calibration quality >85%) proceeded to the experiment, and no participants were excluded for signal quality. The fNIRS data were analyzed using Homer2 software [31], following the processing procedures and parameters outlined in Brigadoi et al. [32]. Channels with oversaturated light intensity were excluded. The relative coefficient of variation (CV, %) was used to identify bad channels, with participants excluded if any channel had a CV >15% [33], no participant met the exclusion criterion. Channels with a CV ≤15% were retained, and bad channels were treated as missing values in the statistical analysis, the missing rate was 10.48%.

The original light intensity data were converted into relative changes of optical density (OD) data for each participant. Motion artifacts in the OD data were identified and corrected using moving standard deviation and cubic spline interpolation [16]. Physiological signals were removed using a low‐pass filter with a cut‐off of 0.2 Hz, and low‐frequency drift was corrected with a high‐pass filter at 0.01 Hz [34]. After filtering, the OD data were converted into Oxy‐Hb concentration data following the Beer–Lambert Law. We focused only on changes in Oxy‐Hb concentration, as it provides a superior signal‐to‐noise ratio and better reflects task‐related cortical activation compared to Deoxy‐Hb [20, 35]. The visualization of Oxy‐Hb concentrations was used BrainNet viewer toolbox [36].

The data were analyzed using linear mixed‐effects modeling (LMM) in R [37]. The significance of model parameters was assessed using the lmerTest package [38]. Fixed effects included group (NA, SA, and CA) and emotional valence (positive, neutral, and negative picture), their interaction, and covariates (age, gender, education level, and depression score). Random effects were estimated for Subject ID.

Type III ANOVA with Satterthwaite’s method for degrees of freedom was conducted on these models using the anova function from the car package [39]. Significance tests were based on Satterthwaite *p*‐values [40], and effect size was computed using partial eta squared (*η* ^2^_*p*_) from the effectsize package [41]. In cases where significant main effects or interactions were observed, follow‐up contrasts were performed using the emmeans package in R [42], with Tukey adjustment for controlling multiple comparisons.

## 3. Results

### 3.1. HADS Scores of Participants

The HADS anxiety scores showed significant differences among the three groups, *F* (2, 839) = 2327.10, *p* < 0.001 (Table 1). Tukey’s HSD post hoc tests indicated that both the SA (*p* < 0.001) and CA (*p* < 0.001) groups had significantly higher HADS anxiety scores than the NA group. Additionally, the CA group had significantly higher scores than the SA group (*p* < 0.001).

### 3.2. Comparison of Hemodynamic Response Across Three Groups

For Channel 30, corresponded to the right frontopolar cortex (rFPC), in terms of Oxy‐Hb levels, the main effect of group was significant, *F* (2, 2466) = 9.56, *p*_FDR_ = 0.004, and *η* ^2^_*p*_ = 0.008. Post hoc test results indicated that the NA (*p*_FDR_ = 0.038) and SA (*p*_FDR_ <0.001) groups had significantly higher compared to the CA group. However, no significant difference was found between the NA and SA groups (*p*_FDR_ = 0.156) (Figure 3a). The main effect of emotional valence was not significant, *F* (2, 2466) = 1.30, *p*_FDR_ = 1.000, *η* ^2^_*p*_ = 0.001, and the interaction effect between group and emotional valence was also not significant, *F* (4, 2466) = 1.07, *p*_FDR_ = 1.000, and *η* ^2^_*p*_ = 0.002.

Figure 3Oxy‐Hb levels among the three groups during EAMT. (a) Channel 30. (b) Channel 33.  ^∗∗∗^ *p* < 0.001. CA, confirmed anxiety group; NA, no anxiety group; SA, suspected anxiety group.

**Figure:**
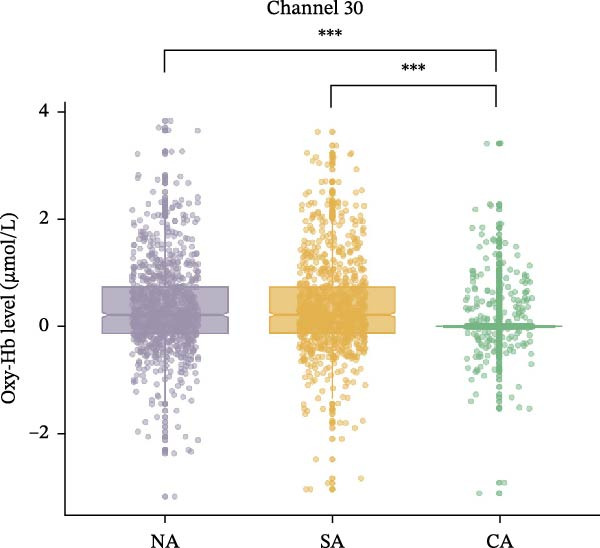
(a)

**Figure:**
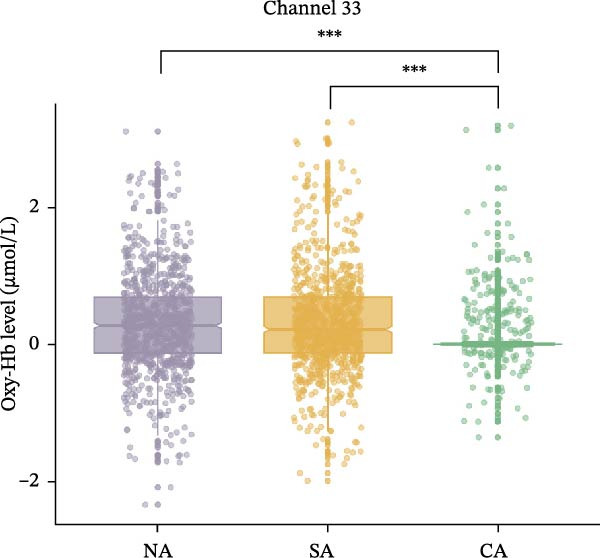
(b)

**Figure 4:**
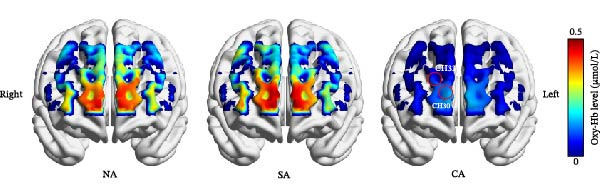
Hemodynamic concentrations among the three groups during EAMT. CA, confirmed anxiety group; CH, channel; NA, no anxiety group; SA, suspected anxiety group.

For Channel 33, corresponded to the rFPC, in terms of Oxy‐Hb levels, the main effect of group was significant, *F* (2, 2455) = 16.46, *p*_FDR_ <0.001, and *η* ^2^_*p*_ = 0.01. Post hoc test results indicated that the NA (*p*_FDR_ < 0.001) and SA (*p*_FDR_ < 0.001) groups had significantly higher compared to the CA group. However, no significant difference was found between the NA and SA groups (*p*_FDR_ = 0.312) (Figure 3b). The main effect of emotional valence was not significant, *F* (2, 2455) = 0.92, *p*_FDR_ = 1.000, $\eta_{p}^{2}\;<0.001$, and the interaction effect between group and emotional valence was also not significant, *F* (4, 2455) = 0.10, *p*_FDR_ = 1.000, *η* ^2^_*p*_ = 0.002. These group‐level differences in Oxy‐Hb concentrations during the EAMT are shown in Figure 4.

### 3.3. Correlation With Changes in Oxy‐Hb Levels and the Anxiety Scores

Partial correlation analysis was conducted to explore the relationship between Oxy‐Hb levels and anxiety scores that showed significant group differences, with age, gender, education level, and depression as the control variable. Channel 30 (*r* = −0.15, *p* < 0.001) (Figure 5a) and channel 33 (*r* = −0.16, *p* < 0.001) (Figure 5b) consistently showed negative correlation between Oxy‐Hb levels and anxiety scores.

Figure 5The scatter plots between Oxy‐Hb concentrations and anxiety score in channels. (a) Channel 30. (b) Channel 33.

**Figure:**
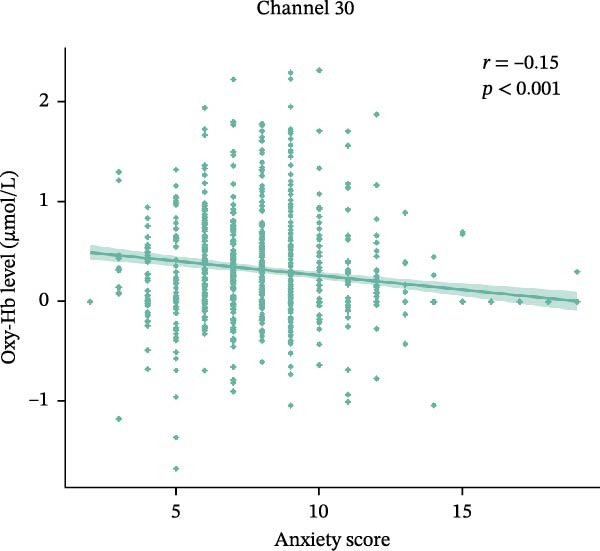
(a)

**Figure:**
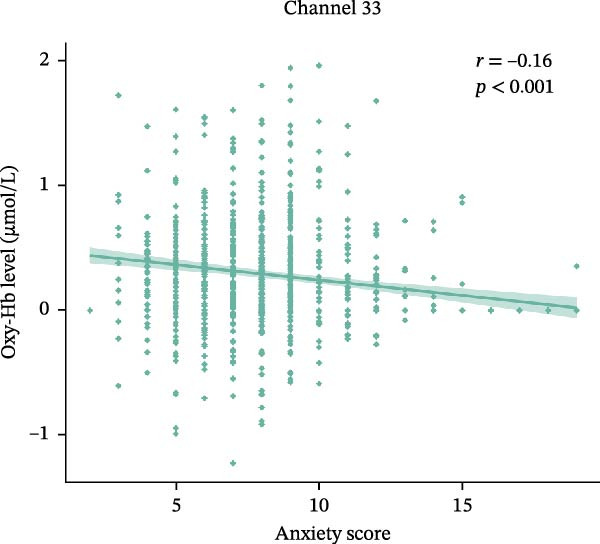
(b)

## 4. Discussion

In this study, fNIRS was used to confirm the difference in activation of the frontal cortex among healthy subjects and patients with SA and CA during EAMT. HADS scores demonstrated a significant stepwise increase across the three groups. This graded anxiety profile provides a foundation for interpreting neurophysiological differences. We also observed group differences in Oxy‐Hb concentrations, with the CA group showing the lowest levels, particularly in the rFPC, where significant differences emerged. Correlation analysis further revealed that more severe anxiety levels were associated with decreased Oxy‐Hb concentrations. These findings describe group‐level prefrontal activation differences associated with anxiety severity. fNIRS may be a valuable tool for characterizing individuals, but its diagnostic utility was not evaluated.

This study revealed no significant difference in Oxy‐Hb across positive, neutral, and negative cues. This pattern aligns with EAMT evidence that anxiety often exhibits valence‐insensitive prefrontal responses. Specifically, anxious depression patients tend to show no differential between positive and negative emotional valence in the dorsolateral PFC (dlPFC), whereas healthy controls and non‐anxious depression showed higher activation to positive than negative cues [24, 43]. Zhang et al. [24] likewise reported dissociable valence sensitivity, pure anxiety exhibits right dlPFC blunting to positive stimuli and pure depression shows reduced sensitivity to negative stimuli. In pure anxiety, fear generalization, uncertainty‐driven sustained anticipation, and a reliable threat‐related attentional bias keep prefrontal control broadly engaged and reduce cue‐specific differentiation, thereby compressing responses across positive, neutral, and negative conditions and yielding a valence‐insensitive pattern [44–46]. Some EAMT studies have found that even in the absence of emotional valence‐specific interactions, lateralized activation in the PFC reliably reflects emotional processing, and proposed that EAMT performance cannot be the gold standard for the diagnosis of anxiety [47, 48]. Thus, our nonsignificant valence effect reflects anxiety group‐specific neurophenotypes.

We found that Oxy‐Hb group differences were most prominent in Channels 30 and 33, both located in the rFPC. Trait‐related functional theories of the PFC propose that the PFC and FPC share common functions such as emotional regulation, cognitive control, and behavioral inhibition, and right PFC is highly relevant to the regulation of negative emotions [49–51]. A review on the lateralization of the PFC in anxiety expression and regulation suggests that the left PFC is primarily sensitive to verbal content, while the right PFC is mainly sensitive to emotional content stimuli [52]. Individuals with lower right PFC activation tend to have greater difficulty recovering from negative affect or stress [53]. Therefore, the lower activation in the right PFC among individuals with CA in this study may reflect a deficiency in emotion regulation. This difference provides region‐specific support for the accurate diagnosis of anxiety. The consistent involvement highlights the right FPC as a promising target for intervention. Future studies should evaluate the efficacy of noninvasive neuromodulation techniques (e.g., TMS, TES, and DCS) in enhancing emotion regulation in anxiety disorders [54, 55].

Across the whole sample, we found no Oxy‐Hb differences between the NA and SA groups, yet both exhibited higher concentrations than the CA group, which showed the lowest level. Consistent with previous fNIRS studies showing that individuals with higher levels of anxiety disorders exhibit lower prefrontal activation—particularly in the rFPC—compared to those with lower anxiety levels or healthy controls [56–58]. This pattern suggests that subclinical anxiety may engage compensatory prefrontal mechanisms to preserve hemodynamic responses, whereas clinically significant anxiety overwhelms these processes, resulting in detectable PFC hypoactivation [59–61]. Therefore, the precise neurobiological boundary between subclinical and clinically significant anxiety remains to be clarified.

Correlation analysis further indicated a negative association between anxiety severity and Oxy‐Hb specifically in the rFPC, consistent with earlier studies linking anxiety to reduced prefrontal activation [62, 63]. The severity of both generalized and pathological anxiety has been found to negatively correlate with PFC activation. In a study involving emotional face recognition, the severity of generalized anxiety was found to be negatively correlated with PFC activation [64]. Large‐sample fNIRS without valence manipulation still detects robust right dlPFC, Broca and bilateral FPC hypoactivation scaling with symptom severity [25]. As the PFC is involved in the integration of emotional information and regulation of emotional responses, its dysfunction may impair emotional processing in individuals with anxiety [51]. As shown in prior research, decreased recruitment of PFC regions leads to poor downregulation of arousal and a shift from adaptive to maladaptive responses under stress [65]. The results support fNIRS as a probe of group‐level anxiety‐related activation, but its diagnostic utility remains untested.

## 5. Limitations and Future Directions

The study also has a few limitations. First, from a methodological perspective, we used only the EAMT and did not incorporate short‐separation channels to filter extracerebral signals, such as scalp blood flow, which may limit the interpretability of the neural data and should be addressed in future research. Second, anxiety was assessed solely using the HADS‐A, future studies should include additional validated scales for broader diagnostic coverage. Third, unequal group sizes may have reduced statistical power and external validity, and future studies should aim for balanced samples. The observed association between anxiety severity and altered Oxy‐Hb may highlight a potential physiological target. Future research should evaluate PFC‐targeted interventions to strengthen emotion regulation in anxiety disorders.

## 6. Conclusion

The present study found that the strongest group effects localized to the rFPC (Channels 30, 33): NA and SA showed higher Oxy‐Hb than CA, and emotional valence exerted no effect. Oxy‐Hb in these channels decreased with greater anxiety severity. These findings indicate a group‐level pattern of rFPC hypoactivation linked to anxiety severity during autobiographical recall. Future studies should incorporate short‐separation channels and broader diagnostic batteries to enhance neural specificity and clinical applicability.

## Author Contributions

Under the supervision of Yan Zhang, Hui Shi, and Marc N. Potenza, Honglin Ren and Qiang Xiao performed the experimental design and data analysis. Honglin Ren and Yajie Wang made significant contributions to writing of the original draft, as well as to review and editing. Delong Zhou, Youcai Yang, and Fuxing Wang contributed to conceptualization, data curation, formal analysis, and methodology.

## Funding

This study was supported and granted by the China Vocational Education Association of Hubei (Grant BHZJ2024326) and the Fundamental Research Funds for the Central Universities: HUST (Grant 2026ZDCG001).

## Disclosure

All the authors have approved the final manuscript for publication.

## Ethics Statement

All procedures performed in studies involving human participants were conducted in accordance with the ethical standards of the institutional and/or national research committee, as well as with the 1964 Helsinki Declaration and its later amendments or comparable ethical standards. This study was performed in accordance with the Declaration of Helsinki and was approved by the Ethics Committee of the School of Huazhong University of Science and Technology (Approval Number: 20190912).

## Conflicts of Interest

The authors declare no conflicts of interest.

## Supporting Information

Additional supporting information can be found online in the Supporting Information section.

## Supporting information


**Supporting Information** Appendix S1. The Montreal Neurological Institute coordinates and the mapping Brodmann area of each channel.

## Data Availability

The data supporting the findings of this study are available from the corresponding author upon reasonable request. Database source: Mental Health and Brain Sciences Laboratory Research Database in School of Education, Huazhong University of Science and Technology.

## References

[1] LeDoux J. E. and Pine D. S. , Using Neuroscience to Help Understand Fear and Anxiety: A Two-System Framework, American Journal of Psychiatry. (2016) 173, no. 11, 1083–1093, 10.1176/appi.ajp.2016.16030353, 2-s2.0-84994112822. 27609244

[2] Kessler R. C. , Berglund P. , Demler O. , Jin R. , Merikangas K. R. , and Walters E. E. , Lifetime Prevalence and Age-of-Onset Distributions of DSM-IV Disorders in the National Comorbidity Survey Replication, Archives of General Psychiatry. (2005) 62, no. 6, 10.1001/archpsyc.62.6.593, 2-s2.0-20344385026, 593. 15939837

[3] Prins M. A. , Verhaak P. F. M. , Bensing J. M. , and van der Meer K. , Health Beliefs and Perceived Need for Mental Health Care of Anxiety and Depression—the Patients’ Perspective Explored, Clinical Psychology Review. (2008) 28, no. 6, 1038–1058, 10.1016/j.cpr.2008.02.009, 2-s2.0-45049085982. 18420323

[4] Rice D. P. and Miller L. S. , Health Economics and Cost Implications of Anxiety and Other Mental Disorders in the United States, British Journal of Psychiatry. (1998) 173, no. S34, 4–9, 10.1192/S0007125000293458. 9829010

[5] Coles M. E. and Heimberg R. G. , Memory Biases in the Anxiety Disorders: Current Status, Clinical Psychology Review. (2002) 22, no. 4, 587–627, 10.1016/S0272-7358(01)00113-1, 2-s2.0-0036239030. 12094512

[6] Ros L. , Romero D. , Ricarte J. J. , Serrano J. P. , Nieto M. , and Latorre J. M. , Measurement of Overgeneral Autobiographical Memory: Psychometric Properties of the Autobiographical Memory Test in Young and Older Populations, PLOS ONE. (2018) 13, no. 4, 10.1371/journal.pone.0196073, 2-s2.0-85045896641, e0196073. 29672583 PMC5908191

[7] Williams J. M. G. , Capture and Rumination, Functional Avoidance, and Executive Control (CaRFAX): Three Processes That Underlie Overgeneral Memory, Cognition and Emotion. (2006) 20, no. 3-4, 548–568, 10.1080/02699930500450465, 2-s2.0-33745115057. 26529222

[8] Williams J. M. and Broadbent K. , Autobiographical Memory in Suicide Attempters, Journal of Abnormal Psychology. (1986) 95, no. 2, 144–149, 10.1037/0021-843X.95.2.144, 2-s2.0-0022916695. 3711438

[9] Mills C. , D’Mello S. , and Gray M. , On the Validity of the Autobiographical Emotional Memory Task for Emotion Induction, PLoS ONE. (2014) 9, no. 4, 10.1371/journal.pone.0095837, 2-s2.0-84899768980, e95837. 24776697 PMC4002425

[10] Strack F. , Schwarz N. , and Gschneidinger E. , Happiness and Reminiscing: The Role of Time Perspective, Affect, and Mode of Thinking, Journal of Personality and Social Psychology. (1985) 49, no. 6, 1460–1469, 10.1037/0022-3514.49.6.1460, 2-s2.0-0001355693.

[11] Etkin A. and Wager T. D. , Functional Neuroimaging of Anxiety: A Meta-Analysis of Emotional Processing in PTSD, Social Anxiety Disorder, and Specific Phobia, The American Journal of Psychiatry. (2007) 164, no. 10, 1476–1488, 10.1176/appi.ajp.2007.07030504, 2-s2.0-35748963237. 17898336 PMC3318959

[12] Holzschneider K. and Mulert C. , Neuroimaging in Anxiety Disorders, Dialogues in Clinical Neuroscience. (2011) 13, no. 4, 453–461, 10.31887/DCNS.2011.13.4/kholzschneider. 22275850 PMC3263392

[13] Wenzel A. and Jordan J. , Autobiographical Memory in Angry and Anxious Individuals, Behaviour Research and Therapy. (2005) 43, no. 8, 1099–1109, 10.1016/j.brat.2005.01.008, 2-s2.0-20444497601. 15967178

[14] Bremner J. D. , Brain Imaging in Anxiety Disorders, Expert Review of Neurotherapeutics. (2014) 4, no. 2, 275–284, 10.1586/14737175.4.2.275, 2-s2.0-1542284146. 15853569

[15] Ferrari M. and Quaresima V. , A Brief Review on the History of Human Functional Near-Infrared Spectroscopy (fNIRS) Development and Fields of Application, NeuroImage. (2012) 63, no. 2, 921–935, 10.1016/j.neuroimage.2012.03.049, 2-s2.0-84866160260. 22510258

[16] Scholkmann F. , Spichtig S. , Muehlemann T. , and Wolf M. , How to Detect and Reduce Movement Artifacts in Near-Infrared Imaging Using Moving Standard Deviation and Spline Interpolation, Physiological Measurement. (2010) 31, no. 5, 649–662, 10.1088/0967-3334/31/5/004, 2-s2.0-77951098827. 20308772

[17] Cyranoski D. , Neuroscience: Thought Experiment, Nature. (2011) 469, no. 7329, 148–149, 10.1038/469148a, 2-s2.0-78651406705. 21228849

[18] Ehlis A.-C. , Schneider S. , Dresler T. , and Fallgatter A. J. , Application of Functional Near-Infrared Spectroscopy in Psychiatry, NeuroImage. (2014) 85, 478–488, 10.1016/j.neuroimage.2013.03.067, 2-s2.0-84889643184. 23578578

[19] Hoshi Y. , Kobayashi N. , and Tamura M. , Interpretation of Near-Infrared Spectroscopy Signals: A Study With a Newly Developed Perfused Rat Brain Model, Journal of Applied Physiology. (2001) 90, no. 5, 1657–1662, 10.1152/jappl.2001.90.5.1657. 11299252

[20] Strangman G. , Culver J. P. , Thompson J. H. , and Boas D. A. , A Quantitative Comparison of Simultaneous BOLD fMRI and NIRS Recordings During Functional Brain Activation, NeuroImage. (2002) 17, no. 2, 719–731, 10.1006/nimg.2002.1227. 12377147

[21] Davidson R. J. , Anxiety and Affective Style: Role of Prefrontal Cortex and Amygdala, Biological Psychiatry. (2002) 51, no. 1, 68–80, 10.1016/S0006-3223(01)01328-2, 2-s2.0-0036153246. 11801232

[22] Zigmond A. S. and Snaith R. P. , The Hospital Anxiety and Depression Scale, Acta Psychiatrica Scandinavica. (1983) 67, no. 6, 361–370, 10.1111/j.1600-0447.1983.tb09716.x, 2-s2.0-0020527558. 6880820

[23] Anderson R. J. , Dewhurst S. A. , and Dean G. M. , Direct and Generative Retrieval of Autobiographical Memories: The Roles of Visual Imagery and Executive Processes, Consciousness and Cognition. (2017) 49, 163–171, 10.1016/j.concog.2017.02.010, 2-s2.0-85013004938. 28214766

[24] Zhang Y. , Li X. , and Guo Y. , et al.Dorsolateral Prefrontal Activation in Emotional Autobiographical Task in Depressed and Anxious College Students: An fNIRS Study, International Journal of Environmental Research and Public Health. (2022) 19, no. 21, 10.3390/ijerph192114335, 14335. 36361214 PMC9657988

[25] Wu H. , Lu B. , Zhang Y. , and Li T. , Differences in Prefrontal Cortex Activation in Chinese College Students With Different Severities of Depressive Symptoms: A Large Sample of Functional Near-Infrared Spectroscopy (fNIRS) Findings, Journal of Affective Disorders. (2024) 350, 521–530, 10.1016/j.jad.2024.01.044. 38237870

[26] Bai L. , Ma H. , Huang Y. , and Luo Y. , The Development of Native Chinese Affective Picture System: A Pretest in 46 College Students, Chinese Mental Health Journal. (2005) 19, no. 11, 719–722.

[27] Okamoto M. , Dan H. , and Sakamoto K. , et al.Three-Dimensional Probabilistic Anatomical Cranio-Cerebral Correlation via the International 10–20 System Oriented for Transcranial Functional Brain Mapping, NeuroImage. (2004) 21, no. 1, 99–111, 10.1016/j.neuroimage.2003.08.026, 2-s2.0-9144223620. 14741647

[28] Singh A. K. , Okamoto M. , Dan H. , Jurcak V. , and Dan I. , Spatial Registration of Multichannel Multi-Subject fNIRS Data to MNI Space Without MRI, NeuroImage. (2005) 27, no. 4, 842–851, 10.1016/j.neuroimage.2005.05.019, 2-s2.0-24944583168. 15979346

[29] Ye J. , Tak S. , Jang K. , Jung J. , and Jang J. , NIRS-SPM: Statistical Parametric Mapping for Near-Infrared Spectroscopy, NeuroImage. (2009) 44, no. 2, 428–447, 10.1016/j.neuroimage.2008.08.036, 2-s2.0-56349151815. 18848897

[30] Zhu W. , Wang F. , Mayer R. E. , and Liu T. , Effects of Explaining a Science Lesson to Others or to Oneself: A Cognitive Neuroscience Approach, Learning and Instruction. (2024) 91, 10.1016/j.learninstruc.2024.101897, 101897.

[31] Huppert T. J. , Diamond S. G. , Franceschini M. A. , and Boas D. A. , HomER: A Review of Time-Series Analysis Methods for Near-Infrared Spectroscopy of the Brain, Applied Optics. (2009) 48, no. 10, 10.1364/AO.48.00D280, 2-s2.0-65249169372, D280. 19340120 PMC2761652

[32] Brigadoi S. , Ceccherini L. , and Cutini S. , et al.Motion Artifacts in Functional Near-Infrared Spectroscopy: A Comparison of Motion Correction Techniques Applied to Real Cognitive Data, NeuroImage. (2014) 85, 181–191, 10.1016/j.neuroimage.2013.04.082, 2-s2.0-84889678991. 23639260 PMC3762942

[33] Piper S. K. , Krueger A. , and Koch S. P. , et al.A Wearable Multi-Channel fNIRS System for Brain Imaging in Freely Moving Subjects, NeuroImage. (2014) 85, 64–71, 10.1016/j.neuroimage.2013.06.062, 2-s2.0-84889644462. 23810973 PMC3859838

[34] Arun K. M. , Smitha K. A. , Sylaja P. N. , and Kesavadas C. , Identifying Resting-State Functional Connectivity Changes in the Motor Cortex Using fNIRS During Recovery from Stroke, Brain Topography. (2020) 33, no. 6, 710–719, 10.1007/s10548-020-00785-2. 32685998

[35] Huppert T. J. , Hoge R. D. , Diamond S. G. , Franceschini M. A. , and Boas D. A. , A Temporal Comparison of BOLD, ASL, and NIRS Hemodynamic Responses to Motor Stimuli in Adult Humans, NeuroImage. (2006) 29, no. 2, 368–382, 10.1016/j.neuroimage.2005.08.065, 2-s2.0-30344470220. 16303317 PMC2692693

[36] Xia M. , Wang J. , He Y. , and Csermely P. , BrainNet Viewer: A Network Visualization Tool for Human Brain Connectomics, PLoS ONE. (2013) 8, no. 7, 10.1371/journal.pone.0068910, 2-s2.0-84879833765, e68910. 23861951 PMC3701683

[37] Bates D. , Mächler M. , Bolker B. , and Walker S. , Fitting Linear Mixed-Effects Models Using lme4, Journal of Statistical Software. (2015) 67, no. 1, 10.18637/jss.v067.i01, 2-s2.0-84943645306.

[38] Kuznetsova A. , Brockhoff P. B. , and Christensen R. H. B. , lmerTest Package: Tests in Linear Mixed Effects Models, Journal of Statistical Software. (2017) 82, no. 13, 10.18637/jss.v082.i13.

[39] Fox J. and Weisberg S. , An R Companion to Applied Regression, 2019, 3rd edition, SAGE.

[40] Luke S. G. , Evaluating Significance in Linear Mixed-Effects Models in R, Behavior Research Methods. (2017) 49, no. 4, 1494–1502, 10.3758/s13428-016-0809-y, 2-s2.0-84987657458. 27620283

[41] Ben-Shachar M. , Lüdecke D. , and Makowski D. , Effectsize: Estimation of Effect Size Indices and Standardized Parameters, Journal of Open Source Software. (2020) 5, no. 56, 10.21105/joss.02815, 2815.

[42] Lenth R. V. and Piaskowski J. , Emmeans: Estimated Marginal Means, Aka Least-Squares Means, 2017, p. 2.0.1, Oct. 20 10.32614/CRAN.package.emmeans.

[43] Wu H. , Lu B. , and Xiang N. , et al.Different Activation in Dorsolateral Prefrontal Cortex Between Anxious Depression and Non-Anxious Depression During an Autobiographical Memory Task: A fNIRS Study, Journal of Affective Disorders. (2024) 362, 585–594, 10.1016/j.jad.2024.07.031. 39019227

[44] Lissek S. , Kaczkurkin A. N. , Rabin S. , Geraci M. , Pine D. S. , and Grillon C. , Generalized Anxiety Disorder Is Associated With Overgeneralization of Classically Conditioned Fear, Biological Psychiatry. (2014) 75, no. 11, 909–915, 10.1016/j.biopsych.2013.07.025, 2-s2.0-84899107404. 24001473 PMC3938992

[45] Grupe D. W. and Nitschke J. B. , Uncertainty and Anticipation in Anxiety: An Integrated Neurobiological and Psychological Perspective, Nature Reviews Neuroscience. (2013) 14, no. 7, 488–501, 10.1038/nrn3524, 2-s2.0-84879369197. 23783199 PMC4276319

[46] Bar-Haim Y. , Lamy D. , Pergamin L. , Bakermans-Kranenburg M. J. , and van IJzendoorn M. H. , Threat-Related Attentional Bias in Anxious and Nonanxious Individuals: A Meta-Analytic Study, Psychological Bulletin. (2007) 133, no. 1, 1–24, 10.1037/0033-2909.133.1.1, 2-s2.0-33846097861. 17201568

[47] Zheng M. , Xiang N. , and Qiu M. , et al.Different Dorsolateral Prefrontal Activation During an Emotional Autobiographical Memory Task Between Male and Female Depressed Individuals: A fNIRS Study, NeuroReport. (2024) 35, no. 18, 1173–1182, 10.1097/WNR.0000000000002112. 39445524

[48] Hoshi Y. , Huang J. , and Kohri S. , et al.Recognition of Human Emotions From Cerebral Blood Flow Changes in the Frontal Region: A Study With Event-Related Near-Infrared Spectroscopy, Journal of Neuroimaging. (2011) 21, no. 2, e94–e101, 10.1111/j.1552-6569.2009.00454.x, 2-s2.0-79953045961. 20002968

[49] Kenwood M. M. , Kalin N. H. , and Barbas H. , Correction: The Prefrontal Cortex, Pathological Anxiety, and Anxiety Disorders, Neuropsychopharmacology. (2022) 47, no. 5, 1141–1141, 10.1038/s41386-021-01216-x. 35110689 PMC8938472

[50] Manelis A. , Huppert T. J. , Rodgers E. , Swartz H. A. , and Phillips M. L. , The Role of the Right Prefrontal Cortex in Recognition of Facial Emotional Expressions in Depressed Individuals: FNIRS Study, Journal of Affective Disorders. (2019) 258, 151–158, 10.1016/j.jad.2019.08.006, 2-s2.0-85070260582. 31404763 PMC6710146

[51] Cabeza R. and Nyberg L. , Imaging Cognition II: An Empirical Review of 275 PET and fMRI Studies, Journal of Cognitive Neuroscience. (2000) 12, no. 1, 1–47, 10.1162/08989290051137585, 2-s2.0-0034054982. 10769304

[52] White L. K. , Makhoul W. , Teferi M. , Sheline Y. I. , and Balderston N. L. , The Role of dlPFC Laterality in the Expression and Regulation of Anxiety, Neuropharmacology. (2023) 224, 10.1016/j.neuropharm.2022.109355, 109355. 36442650 PMC9790039

[53] Hulvershorn L. A. , Karne H. , and Gunn A. D. , et al.Neural Activation During Facial Emotion Processing in Unmedicated Bipolar Depression, Euthymia, and Mania, Biological Psychiatry. (2012) 71, no. 7, 603–610, 10.1016/j.biopsych.2011.10.038, 2-s2.0-84858229181. 22206876 PMC3703667

[54] Guo B. , Zhang M. , Hao W. , Wang Y. , Zhang T. , and Liu C. , Neuroinflammation Mechanisms of Neuromodulation Therapies for Anxiety and Depression, Translational Psychiatry. (2023) 13, no. 1, 10.1038/s41398-022-02297-y. PMC9829236 36624089

[55] Kar S. K. and Sarkar S. , Neuro-Stimulation Techniques for the Management of Anxiety Disorders: An Update, Clinical Psychopharmacology and Neuroscience. (2016) 14, no. 4, 330–337, 10.9758/cpn.2016.14.4.330, 2-s2.0-84992523444. 27776384 PMC5083940

[56] Shen Y. , Wu B. , Yu J. , Mou L. , Wang Z. , and Shen X. , Functional Near-Infrared Spectroscopy (fNIRS) in Patients With Major Depressive Disorder, Generalized Anxiety Disorder and Their Comorbidity: Comparison With Healthy Controls, Asian Journal of Psychiatry. (2025) 105, 10.1016/j.ajp.2025.104382, 104382. 39933260

[57] Li J. , Zhong Y. , and Ma Z. , et al.Emotion Reactivity-Related Brain Network Analysis in Generalized Anxiety Disorder: A Task fMRI Study, BMC Psychiatry. (2020) 20, no. 1, 10.1186/s12888-020-02831-6, 429. 32878626 PMC7466835

[58] Tomita N. and Kumano H. , Self-Focused Attention Related to Social Anxiety During Free Speaking Tasks Activates the Right Frontopolar Area, Current Psychology. (2023) 42, no. 12, 10310–10323, 10.1007/s12144-021-02319-w.

[59] Comte M. , Cancel A. , and Coull J. T. , et al.Effect of Trait Anxiety on Prefrontal Control Mechanisms During Emotional Conflict, Human Brain Mapping. (2015) 36, no. 6, 2207–2214, 10.1002/hbm.22765, 2-s2.0-84928762523. 25664956 PMC6869046

[60] Basten U. , Stelzel C. , and Fiebach C. J. , Trait Anxiety Modulates the Neural Efficiency of Inhibitory Control, Journal of Cognitive Neuroscience. (2011) 23, no. 10, 3132–3145, 10.1162/jocn_a_00003, 2-s2.0-84860390507. 21391763

[61] Zhao Q. , Wang Z. , and Yang C. , et al.Anxiety Symptoms Without Depression Are Associated With Cognitive Control Network (CNN) Dysfunction: An fNIRS Study, Psychophysiology. (2024) 61, no. 7, 10.1111/psyp.14564. 38487932

[62] Wang D. , Lin B. , and Huang Y. , et al.Exploring Neural Correlates Between Anxiety and Inhibitory Ability: Evidence From Task-Based fNIRS, Depression and Anxiety. (2024) 2024, no. 1, 10.1155/2024/8680134, 8680134. 40226748 PMC11918997

[63] Bishop S. J. , Trait Anxiety and Impoverished Prefrontal Control of Attention, Nature Neuroscience. (2009) 12, no. 1, 92–98, 10.1038/nn.2242, 2-s2.0-58149127190. 19079249

[64] Monk C. S. , Nelson E. E. , and McClure E. B. , et al.Ventrolateral Prefrontal Cortex Activation and Attentional Bias in Response to Angry Faces in Adolescents With Generalized Anxiety Disorder, American Journal of Psychiatry. (2006) 163, no. 6, 1091–1097, 10.1176/ajp.2006.163.6.1091, 2-s2.0-85047695174. 16741211

[65] Gallagher M. W. , Bentley K. H. , and Barlow D. H. , Perceived Control and Vulnerability to Anxiety Disorders: A Meta-Analytic Review, Cognitive Therapy and Research. (2014) 38, no. 6, 571–584, 10.1007/s10608-014-9624-x, 2-s2.0-84939896878.

