# Supplementary material for: Anxiety Suppressed Prefrontal Cortex Brain Activity: Insights From a Large Sample of Functional Near‐Infrared Spectroscopy (fNIRS) Data
Source: Depress Anxiety. 2026 Mar 3;2026:9910013. doi: 10.1155/da/9910013 (PMC12956378; doi:10.1155/da/9910013)
Supplement: Supplementary file 1 — Supporting Information Appendix S1. The Montreal Neurological Institute coordinates and the mapping Brodmann area of each channel. [file DA-2026-9910013-s001.docx]

# Anxiety Suppressed Prefrontal Cortex Brain Activity: Insights from a Large Sample of Functional Near-Infrared Spectroscopy (fNIRS) Data

Supplementary files

Appendix S1 page 2-3

Appendix S1 The Montreal Neurological Institute coordinates and the mapping Brodmann area of each channel

| Channel | MNI | | | Brodmann | Percentage of Overlap |
| --- | --- | --- | --- | --- | --- |
|  | x | y | z |  |  |
| CH01 | -57 | 6 | 40 | 6 - Pre-Motor and Supplementary Motor Cortex | 0.74 |
| CH02 | -60 | 19 | 18 | 44 - pars opercularis, part of Broca's area | 0.54 |
| CH03 | -56 | 25 | 26 | 45 - pars triangularis Broca's area | 0.58 |
| CH04 | -50 | 12 | 47 | 6 - Pre-Motor and Supplementary Motor Cortex | 0.41 |
| CH05 | -51 | 30 | 33 | 45 - pars triangularis Broca's area | 0.65 |
| CH06 | -45 | 32 | 41 | 9 - Dorsolateral prefrontal cortex | 0.34 |
| CH07 | -55 | 36 | 4 | 45 - pars triangularis Broca's area | 0.97 |
| CH08 | -52 | 41 | 12 | 45 - pars triangularis Broca's area | 0.90 |
| CH09 | -47 | 52 | 0 | 46 - Dorsolateral prefrontal cortex | 0.92 |
| CH10 | -45 | 12 | 56 | 6 - Pre-Motor and Supplementary Motor Cortex | 0.54 |
| CH11 | -39 | 31 | 48 | 9 - Dorsolateral prefrontal cortex | 0.92 |
| CH12 | -30 | 28 | 56 | 8 - Includes Frontal eye fields | 0.71 |
| CH13 | -46 | 47 | 21 | 45 - pars triangularis Broca's area | 0.62 |
| CH14 | -40 | 49 | 30 | 46 - Dorsolateral prefrontal cortex | 0.74 |
| CH15 | -40 | 59 | 9 | 46 - Dorsolateral prefrontal cortex | 0.54 |
| CH16 | -32 | 62 | 19 | 10 - Frontopolar area | 0.52 |
| CH17 | -31 | 49 | 38 | 9 - Dorsolateral prefrontal cortex | 0.53 |
| CH18 | -20 | 46 | 47 | 9 - Dorsolateral prefrontal cortex | 1.00 |
| CH19 | -21 | 62 | 29 | 10 - Frontopolar area | 0.53 |
| CH20 | -12 | 59 | 40 | 9 - Dorsolateral prefrontal cortex | 0.88 |
| CH21 | -33 | 66 | -2 | 10 - Frontopolar area | 0.52 |
| CH22 | -22 | 71 | 9 | 10 - Frontopolar area | 0.91 |
| CH23 | -13 | 73 | -2 | 10 - Frontopolar area | 0.57 |
| CH24 | -11 | 41 | 56 | 8 - Includes Frontal eye fields | 0.60 |
| CH25 | 0 | 51 | 45 | 9 - Dorsolateral prefrontal cortex | 0.97 |
| CH26 | 12 | 42 | 56 | 8 - Includes Frontal eye fields | 0.63 |
| CH27 | -13 | 71 | 18 | 10 - Frontopolar area | 1.00 |
| CH28 | 1 | 63 | 28 | 10 - Frontopolar area | 0.93 |
| CH29 | 0 | 68 | 8 | 10 - Frontopolar area | 1.00 |
| CH30 | 13 | 71 | 19 | 10 - Frontopolar area | 1.00 |
| CH31 | 12 | 59 | 39 | 9 - Dorsolateral prefrontal cortex | 0.87 |
| CH32 | 21 | 47 | 49 | 9 - Dorsolateral prefrontal cortex | 0.98 |
| CH33 | 22 | 63 | 29 | 10 - Frontopolar area | 0.59 |
| CH34 | 31 | 49 | 39 | 9 - Dorsolateral prefrontal cortex | 0.69 |
| CH35 | 13 | 73 | -2 | 10 - Frontopolar area | 0.62 |
| CH36 | 24 | 72 | 9 | 10 - Frontopolar area | 0.89 |
| CH37 | 34 | 67 | -2 | 11 - Orbitofrontal area | 0.55 |
| CH38 | 30 | 29 | 57 | 8 - Includes Frontal eye fields | 0.80 |
| CH39 | 40 | 31 | 48 | 9 - Dorsolateral prefrontal cortex | 0.93 |
| CH40 | 47 | 12 | 56 | 9 - Dorsolateral prefrontal cortex | 0.59 |
| CH41 | 33 | 64 | 19 | 10 - Frontopolar area | 0.65 |
| CH42 | 41 | 50 | 29 | 46 - Dorsolateral prefrontal cortex | 0.77 |
| CH43 | 43 | 60 | 9 | 10 - Frontopolar area | 0.54 |
| CH44 | 49 | 48 | 22 | 45 - pars triangularis Broca's area | 0.58 |
| CH45 | 46 | 33 | 43 | 9 - Dorsolateral prefrontal cortex | 0.51 |
| CH46 | 53 | 31 | 34 | 45 - pars triangularis Broca's area | 0.69 |
| CH47 | 52 | 12 | 48 | 9 - Dorsolateral prefrontal cortex | 0.48 |
| CH48 | 48 | 55 | 0 | 46 - Dorsolateral prefrontal cortex | 0.87 |
| CH49 | 54 | 42 | 13 | 45 - pars triangularis Broca's area | 0.83 |
| CH50 | 58 | 36 | 4 | 45 - pars triangularis Broca's area | 0.97 |
| CH51 | 58 | 27 | 26 | 45 - pars triangularis Broca's area | 0.69 |
| CH52 | 60 | 6 | 42 | 6 - Pre-Motor and Supplementary Motor Cortex | 0.79 |
| CH53 | 62 | 20 | 19 | 44 - pars opercularis, part of Broca's area | 0.49 |

Abbreviations: MNI: The Montreal Neurological Institute; CH: channel.
